# A rare case of *TFEB*/6p21/*VEGFA*-amplified renal cell carcinoma diagnosed by whole-exome sequencing: clinicopathological and genetic feature report and literature review

**DOI:** 10.1186/s13000-024-01476-3

**Published:** 2024-05-10

**Authors:** Ruiqi Zhang, Meili Ding, Xingyao Zhu, Xiang Li, Qi Hu, Lin Tao, Wenhao Hu, Hong Zou

**Affiliations:** 1 Department of Pathology, The Second Affiliated Hospital of Zhejiang University School of Medicine, Zhejiang, 310009 China; 2 Department of Pathology, The Yangxin County People’s Hospital, Binzhou, 251800 China; 3 Department of Pathology, People’s Hospital of Xinjiang Uygur Autonomous Region, Xinjiang, 830001 China; 4 Department of Pathology, The First Affiliated Hospital, Shihezi University School of Medicine, Xinjiang, 832002 China

**Keywords:** *TFEB*/6p21/*VEGFA*-amplified renal cell carcinoma, Whole exome sequencing, Differential diagnosis of molecular genetic changes

## Abstract

**Background:**

*TFEB*/6p21/*VEGFA*-amplified renal cell carcinoma (RCC) is rare and difficult to diagnose, with diverse histological patterns and immunohistochemical and poorly defined molecular genetic characteristics.

**Case presentation:**

We report a case of a 63-year-old male admitted in 2017 with complex histomorphology, three morphological features of clear cell, eosinophilic and papillary RCC and resembling areas of glomerular and tubular formation. The immunophenotype also showed a mixture of CD10 and P504s. RCC with a high suspicion of collision tumors was indicated according to the 2014 WHO classification system; no precise diagnosis was possible. The patient was diagnosed at a different hospital with poorly differentiated lung squamous cell carcinoma one year after RCC surgery. We exploited molecular technology advances to retrospectively investigate the patient’s molecular genetic alterations by whole-exome sequencing. The results revealed a 6p21 amplification in *VEGFA* and *TFEB* gene acquisition absent in other RCC subtypes. Clear cell, papillary, chromophobe, *TFE3*-translocation, eosinophilic solid and cystic RCC were excluded. Strong TFEB and Melan-A protein positivity prompted rediagnosis as *TFEB*/6p21/*VEGFA*-amplified RCC as per 2022 WHO classification. TMB-L (low tumor mutational load), *CCND3* gene acquisition and *MRE11A* and *ATM* gene deletion mutations indicated sensitivity to PD-1/PD-L1 inhibitor combinations and the FDA-approved targeted agents Niraparib (Grade C), Olaparib (Grade C), Rucaparib (Grade C) and Talazoparib (Class C). GO (Gene Ontology) and KEGG enrichment analyses revealed major mutations and abnormal CNVs in genes involved in biological processes such as the TGF-β, Hippo, E-cadherin, lysosomal biogenesis and autophagy signaling pathways, biofilm synthesis cell adhesion substance metabolism regulation and others. We compared *TFEB*/6p21/*VEGFA*-amplified with *TFEB*-translocated RCC; significant differences in disease onset age, histological patterns, pathological stages, clinical prognoses, and genetic characteristics were revealed.

**Conclusion:**

We clarified the patient’s challenging diagnosis and discussed the clinicopathology, immunophenotype, differential diagnosis, and molecular genetic information regarding *TFEB*/6p21/*VEGFA*-amplified RCC via exome analysis and a literature review.

**Supplementary Information:**

The online version contains supplementary material available at 10.1186/s13000-024-01476-3.

## Highlights

1. For the first time, the molecular genetics of *TFEB*/6p21/*VEGFA*-amplified renal cell carcinoma were completely and systematically characterized by exon sequencing.

2. The first case of *TFEB*/6p21/*VEGFA*-amplified renal cell carcinoma with genomic instability was reported, presenting a new outlook on the treatment and prognosis of this tumor.

3. A systematic review and differentiation of *TFEB*/6p21/*VEGFA*-amplified renal cell carcinoma and *TFEB*-translocated renal cell carcinoma in clinicopathological, histological, immunophenotypic, and molecular genetic features was performed.

**Supplementary Information:**

The online version contains supplementary material available at 10.1186/s13000-024-01476-3.

## Background

*TFEB*/6p21/*VEGFA*-amplified renal cell carcinoma is a rare subtype of renal cell carcinoma that was first proposed as a separate subtype by Argani et al. in 2016 (ref. [1, 2]) and was not included in the WHO until 2022 due to its unique and rare nature. The interpretation of this tumor is imprecise; it is described as a relatively rare and highly aggressive tumor with a specific rate of recurrence and metastasis that tends to occur in middle-aged and older adults [3, 4]. The tissue morphology of the tumor is diverse, mostly resembling papillary renal cell carcinoma (PRCC) with clear cell renal cell carcinoma (CCRCC)- or chromophobe renal cell carcinoma (CHRCC)-like morphology. These tumors demonstrate similar immunohistochemistry results to *TFEB* translocation renal cell carcinoma, commonly expressing pigment differentiation-related markers (Melan-A, HMB45, and cathepsin k). Molecular genetics suggests the presence of altered polyploid amplification in the region where the *TFEB* gene is located (6p21 region), including amplification of the critical genes *VEGFA* and *CCND3*, suspected to be highly associated with the aggressive clinical course of this tumor in the absence of *TFEB* gene translocations [5, 6].

The rare case we report with a mixture of clear cell carcinoma, eosinophilic carcinoma, and papillary renal carcinoma morphology phenotypes and characteristics was found in 2017 and initially diagnosed with renal cell carcinoma by regular morphology and immunohistochemistry analyses due to the limited molecular pathology available at the time [7, 8]. Collision tumor was highly suspected in this patient; one year later, he developed poorly differentiated squamous cell carcinoma of the lung. As second-generation sequencing methods had matured, we continued evaluating this case by whole-exome sequencing and obtained hints of diagnostic value after obtaining in-depth mining sequencing results. Then, through immunohistochemical analysis and an extensive literature review, we differentiated the patient’s tumor from various types of renal cancer and diagnosed it as *TFEB*/6p21/*VEGFA*-amplified renal cell carcinoma. The in-depth analysis of the molecular genetic changes in this case combined with a literature review to explore the relationships of these changes with diagnosis, prognosis, treatment and differential diagnosis with *TFEB* translocation renal cell carcinoma deepens our understanding of such tumors.

## Case presentation

**Fig. 1:**
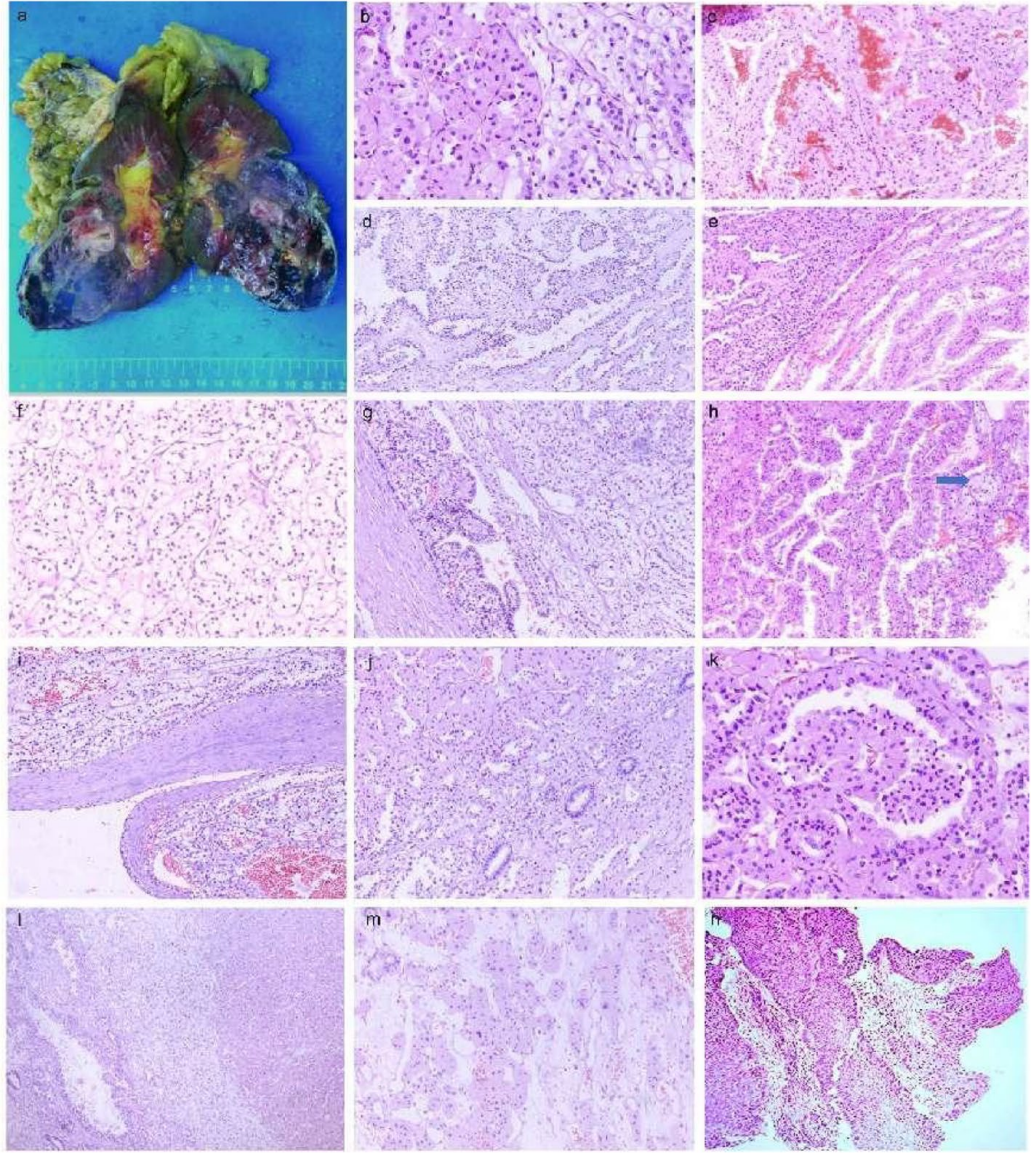
Gross images of the patient and HE staining of the tumor tissue. **a** The left kidney and surrounding fatty tissue were sent for examination, with a total size of 19 × 13 × 7 cm, and the size of the incised kidney was 13.5 × 8 × 6 cm. Most areas of the perirenal fat capsule were easy to peel off, and the focal renal epithelium was adherent to the adipose tissue, with a multicolored appearance and partial dark red necrosis. No lymph nodes were detected in the fatty tissue at the renal hilum. **b** The tumor tissue was biphasic, with areas of eosinophilic and clear cell coexistence. **c** Hemorrhagic and necrotic areas. **d**, **e** The tumor cells are arranged in a nested papillary pattern, and the papillae have a slender fibrovascular axis. **f** Tumor cells had abundant cytoplasm and clear cytoplasm. **g** The tumor cells were arranged in a striated papillary pattern. **h** Foam cell. **i** A fibrous pseudoenvelope is seen around the tumor. **J**, **k** Pseudo papillae and similar glomerular and tubular-like structures. **l** The tumor tissue was biphasic, with areas of eosinophilic and clear cell coexistence. **m** Tumor cells have abundant cytoplasm and eosinophilic cytoplasm. **n** Poorly differentiated squamous cell carcinoma of the lung

A 63-year-old male, was admitted to the hospital for right-sided low back pain in 2017. Fatty liver and a solid mass of the left kidney (internal partial liquefaction) were shown by abdominal ultrasonography, and a space-occupying lesion in the middle and lower part of the left kidney was observed by urinary CT, suggesting the possibility of renal carcinoma. A CT scan of the right kidney, bilateral ureters, and bladder showed no definite abnormal changes, though the rectal wall was slightly thickened. Lung CT showed no obvious abnormality. After admission, the patient underwent laparoscopic radical resection of left renal cancer under general anesthesia, and the operation went smoothly. The patient was diagnosed with poorly differentiated lung squamous cell carcinoma one year after RCC surgery, as shown in Fig. 1h. After receiving two cycles of the "Docetaxel + Cis-platinum + Endo star" systemic intravenous chemotherapy regimen,the patient died. The postoperative survival time of patients with renal cancer was less than three years.

**Fig. 2:**
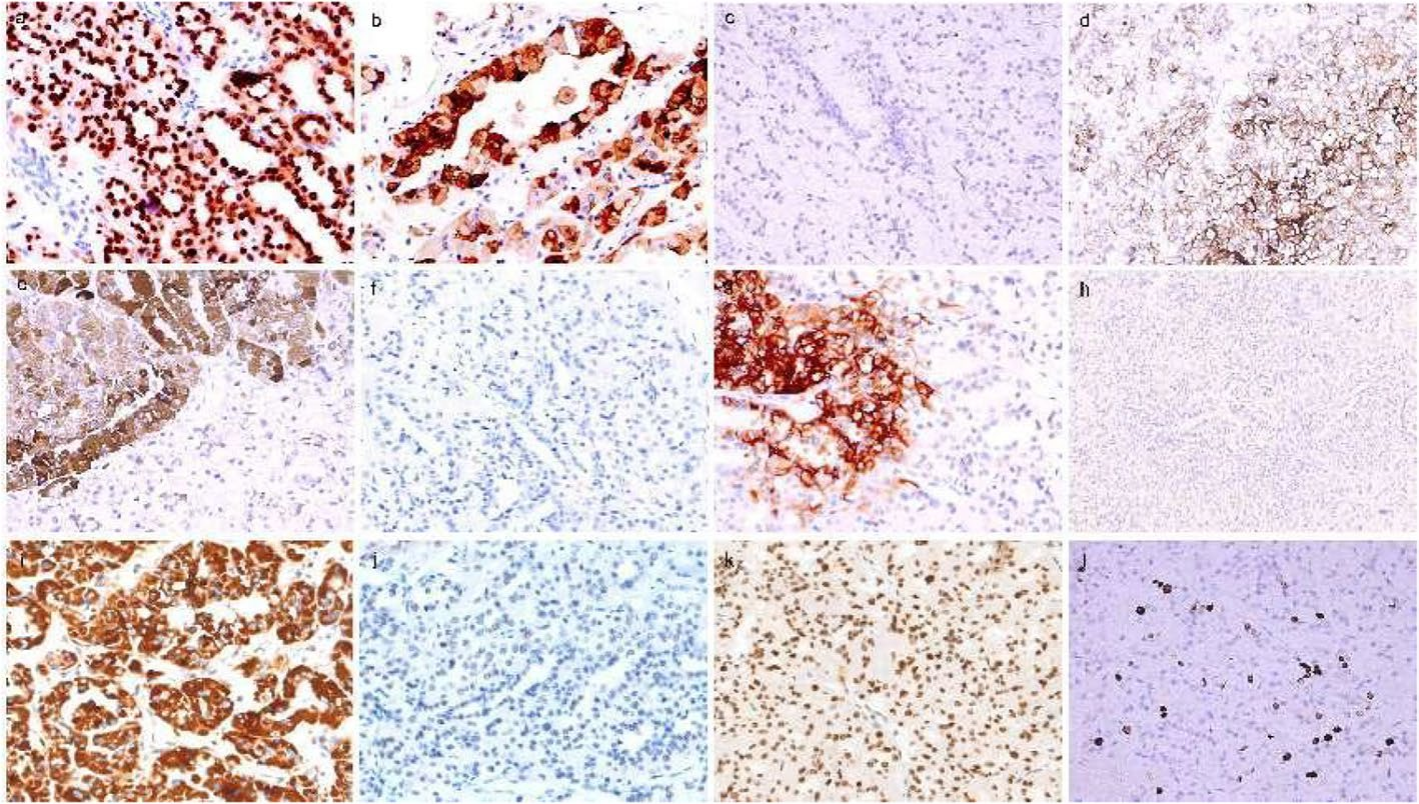
Immunohistochemistry results. **a** Tumor cell nuclei were strongly positive for TFEB × 20 (**b**) Melan-A positive × 20 (**c**) TFE3 negative × 20 (**d**) CD10( +) positive in the clear cell area × 20 (**e**) AMACR diffuse positive in the papillary carcinoma area × 20 (**f**) CD117 negative × 20 (**g**) CA9 focally positive with cancer cells positive in clear differentiation areas and negative in tubular differentiation areas × 20 (**h**) CK7 negative × 20 (**i**) SDHB positive × 20 (**j**) MSH6 negative × 20 and (**k**) MSH2 positive × 20 (**l**) The proliferation index of Ki67 was less than 10% × 20

One left kidney with its surrounding adipose tissue was sent for examination, with a total size of 19 × 13 × 7 cm, and the kidney was dissected to a length of 13.5 × 8 × 6 cm. Most areas of the perirenal fat capsule were easy to peel off, and the focal renal epithelium adhered to the fatty tissue. A mass of 8.3 × 5.8 × 6 cm in size was seen in the middle and lower poles of the kidney, with a colorful external appearance, partly dark red necrosis, partially protruding into the renal pelvis, with a sebaceous thickness of 0.5 cm, and a medullary thickness of 2.8 cm. The ureter was 5 cm long and 0.4–0.5 cm in length. No lymph nodes were detected in the adipose tissue at the renal hilum (Fig. 1a). Microscopically, a fibrous pseudocapsule was observed around the tumor (Fig. 1i), and the tumor cells had a complex composition and diverse morphology (Fig. 1b, l). Some cells were typical of clear cell carcinoma with nested and tubular distribution (Fig. 1d), and some cells resembled eosinophilic papillary carcinoma with a fine fibrous vascular axis in the papilla (Fig. 1e). Foam cells were observed in the focal interstitium (Fig. 1h). In addition, pseudopapillaries and structures resembling glomeruli and renal tubules (Fig. 1j, k) were observed, shift areas were observed in clear cells and the papillary regions, hemorrhage and necrosis were observed in some areas (Fig. 1c), and focal interstitial edema was observed. There was no prominent cell atypia, and mitosis was rare. PAX-8 ( +) and AE1/3 (focal +) were positively expressed in the tumor cells overall, and CD10 ( +) (Fig. 2d) and CA9 (focal + , cancer cells were positive in the clear differentiation area and negative in the tubular differentiation area) were positively expressed in the clear cell area (Fig. 2g). CD31 staining showed strong positive epithelial AMACR (diffuse +) in the papillary carcinoma area except for in the clear cell area with more abundant interstitial vessels (Fig. 2e). There was no loss of SDHB expression in the tumor cells (Fig. 2i). The cells were all negative for CK20, TFE3 (Fig. 2c), CD117 (Fig. 2f), and CK7 (Fig. 2h), and the tumor and had a low Ki-67 proliferation index of approximately 3–5% (Fig. 2l). RCC with a high suspicion of collision tumors was indicated according to the 2014 WHO classification system; no precise diagnosis was possible.

**Fig. 3:**
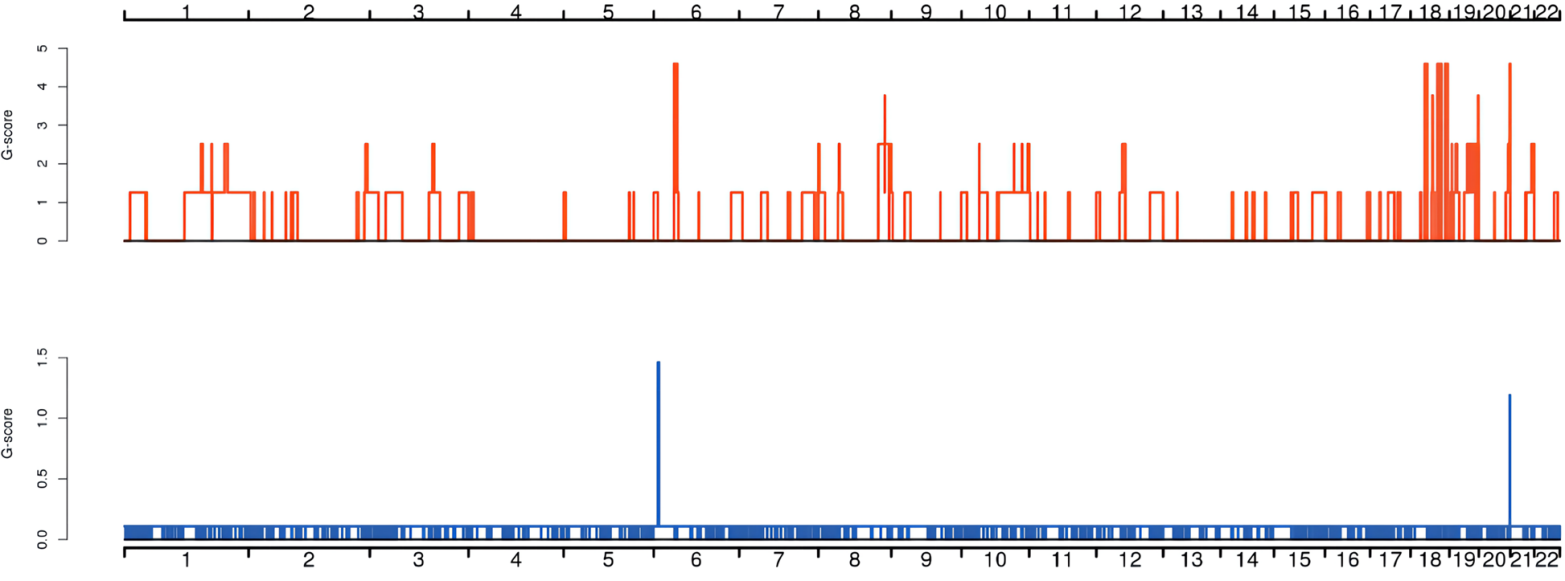
Distribution diagram of high-frequency CNV. The horizontal coordinates are chromosomes 1–22, and sex chromosomes were not considered in this analysis. The vertical coordinates indicate the scores of high-frequency CNV segments by GISTIC software, and higher scores indicate a higher frequency of CNV in this segment. Red indicates an increase in copy number, and blue indicates a decrease in copy number

To determine the molecular genetic alterations in the tumor, we extracted DNA from the patient’s normal tissue and parafn-embedded tumor tissue, performed exon sequencing in 2020. The summary of global mutations in the molecular genetics of this patient was shown in Supplementary Table 1. Given the mutational advantage of CNV in cancer species and overall characteristics, high-frequency CNV analysis was performed on samples to obtain diagnostic information, as shown in Fig. 3. CNVs were concentrated on chromosomes 6, 18, 19, and 21, and the patient demonstrated six significant regions of acquisition, including 6p21.1, 6p12.3, 18q12.1-18q23, 19p13.2, 19q13.2, 19q13.31 and six critical areas of deletion, including 6p21.1–21.3, 6p22.1–22.3, 11q11-11q25, 11p11-11p13, 17q25.1–25.3, and 18q12.1-18q23. Amplification of *TFEB*, *VEGFA*, and *CCND3* genes located on the chromosome 6p21.1 segment (amplification fold > 2) was present, and the *E2F3* gene was lost on the chromosome 6p22.3 segment. The somatic copy number variation (SCNA) characteristics of this patient were further combined and compared with classical oncogenes to find significantly associated driver genes. The *DCC* tumor suppressor gene was absent at 45,100,000–50460000 on chromosome 18. Genetic abnormalities associated with prognosis and treatment shows that the patient had TMB-L (low tumor mutation burden). The amplification mutation of *CCND3* in somatic mutations suggested that the patient would be relatively sensitive to abemaciclib (grade D), palbociclib (grade D), and ribociclib (grade D). The *MRE11A* deletion mutation suggested relative sensitivity to niraparib (grade C), olaparib (grade C), rucaparib (grade C), and talazoparib (grade C). The *ATM* deletion mutation indicated relative sensitivity to Niraparib (grade C), Olaparib (grade C), Rucaparib (grade C), and Talazoparib (grade C) (Table 1).

**Table 1:** Summary of key molecular genetic changes in this patient

	Gene Name		Transcript	Bases change		AA Change
Somatic mutation (Point mutations, insertions, and deletions of small fragments)	*MEN1*		NM_ 130,799	c. 1177C > T		p. Q393
	*NOTCH2*		NM_024408	c.3800A > G		p. E1267G
	*ATOH8*		NM_032827	c.703_711del		p.235_237del
	*ASCC1*		NM_001198799	c.870_878del		p.290_293del
	*DOPEY2*		NM_005128	c.5210_5221del		p. 1737-141del
	*HIST2H2AC*		NM_003517	c.245_258del		p. R82fs
	*APC*		NM_000038	c.4323_4324delAC		p. P1442Sfs 12
	*ZCWPW1*		NM_017984	c.579delC		p. P193fs
	*POU2F3*		NM_001244682	c.350delC		p. T117fs
	*CTC1*		NM_025099	c.2370delC		p. D790fs
	*EXOC1*		NM_001024924	c. 1811dupT		p. I604fs
	*SLC5A12*		NM_ 178,498	c.251_252insA		p. F84fs
	*ATP12A*		NM_001185085	c. 1768 T > A		p. Y590N
	*MNX1*		NM_00551	c. 1154C > A		p. S385
	Gene Name		Transcript	Mutation type		Copy coefficient
Copy number variation	*CCND3*		NM_001760	Gain		7.8
	*VEGFA*		NM_001171623	Gain		4.8
	*SERPINB3*		NM_006919	Gain		4.7
	*SERPINB4*		NM_002974	Gain		4.6
	*BCL2*		NM_000633	Gain		4.4
	*MRE11A*		NM_005591	Loss		0.5
	*ATM*		NM_000051	Loss		0.5
	Gene Name	Chromosome	Position	Ref_ Allele	Alt	Variant_Classification
Cancer Predisposing genes	*PTPRB*	12	70,963,641	C	A	Missense_Mutation
	*IDH2*	15	90,628,130	T	C	Missense_Mutation
	*CACNA1D*	3	53,760,987	G	A	Missense_Mutation
	*AHNAK*	11	62,285,672	A	G	Missense_Mutation
	*PTPRK*	6	128,330,323	A	G	Missense_Mutation
	*RHBDF2*	17	74,475,849	G	A	Missense_Mutation
	*TP53*	17	7,574,012	C	T	Missense_Mutation
Driver mutation	*ARID1B*	6	157,528,016	G	T	Missense_Mutation
	*MAX*	14	65,482,404	A	T	Missense_Mutation
	*NOTCH2*	1	120,471,691	T	C	Missense_Mutation
	*APC*	5	112,175,612	CCA	C	Frame_Shift_Del
	Gene Name	Mutation type	Copy coefficient	Sensitive drugs		
Target drug-related genes	*CCND3*	Gain	7.8	Abemaciclib (Grade D) Palbociclib (Grade D)、Ribociclib(Grade D)		
	*MRE11A*	Loss	0.5	Niraparib(Grade C)、Olaparib(Grade C)、Rucaparib(Grade C)、Talazoparib(Grade C)		
	*ATM*	Loss	0.5	Niraparib(Grade C)、Olaparib(Grade C)、Rucaparib(Grade C)、Talazoparib(Grade C)		

Grade A: FDA approval, or from professional clinical guidelines

Grade B: Confirmed by large-scale clinical studies and consensus of clinical experts

Grade C: Class A evidence in other cancer types or has been used as a screening inclusion criteria in clinical trials, or is supported by multiple small studies

Grade D: Support from preclinical studies or case reports

**Fig. 4:**
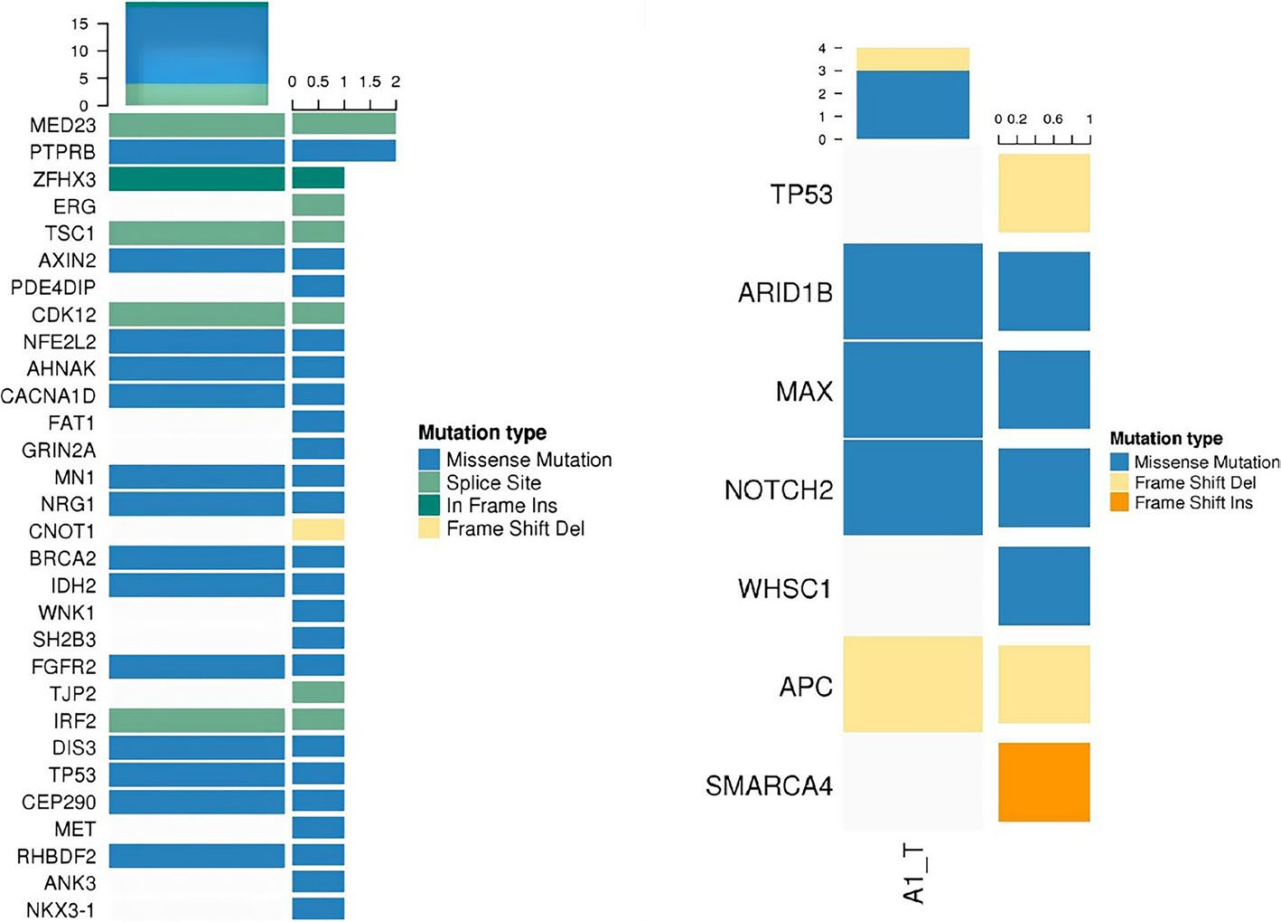
**a** Landscape map of susceptibility genes. **b** Landscape of known driver genes

**Fig. 5:**
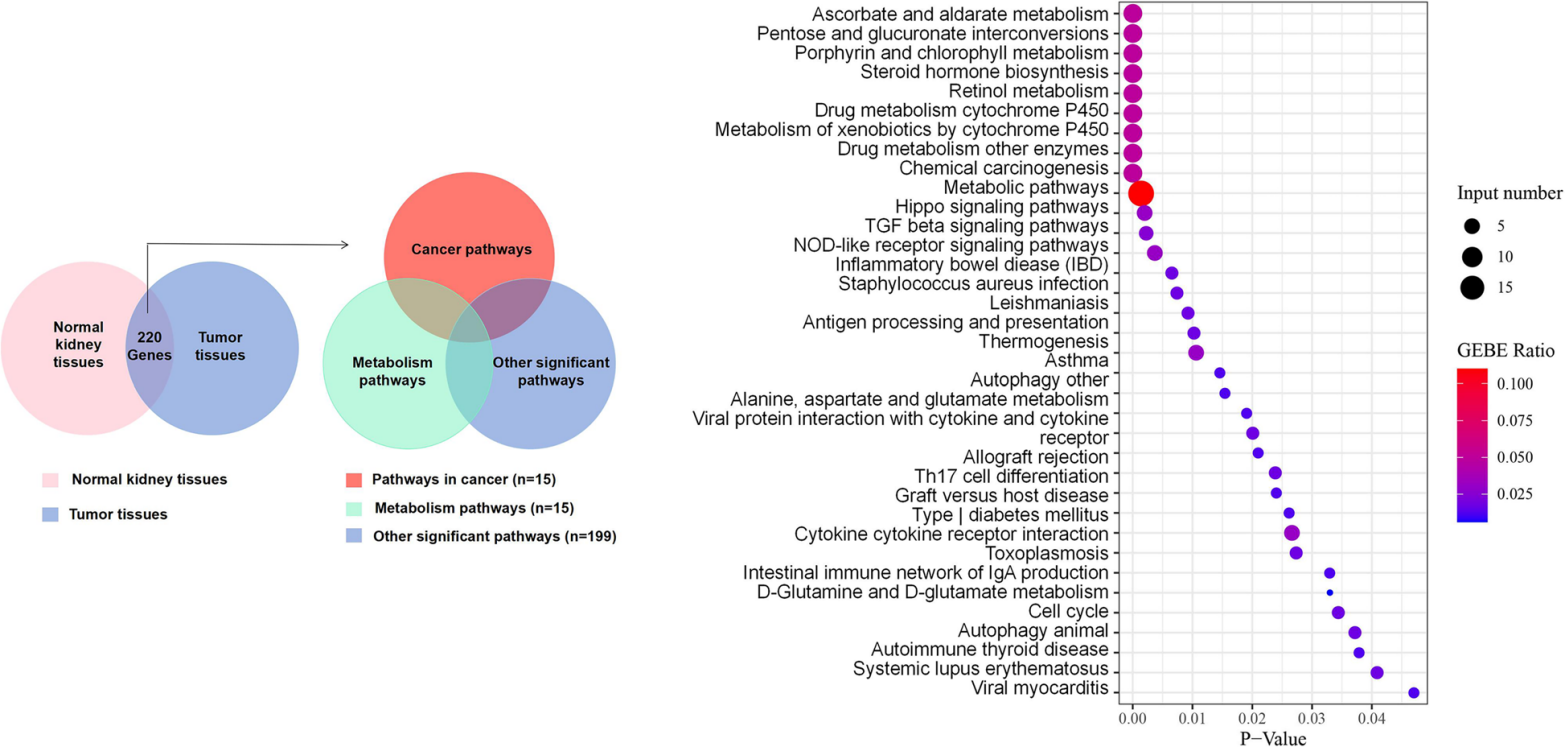
**a** Venn diagram of differentially expressed genes with missense mutations in SNPs of tumor samples versus normal samples. **b** Major pathways involved in SNPs differ between tumor tissue and control tissue in this patient

To further understand the molecular genetic abnormalities of patients, germline mutations were screened by combining SNP comparisons with normal tissues to derive possible tumor susceptibility genes, as shown in Fig. 4a (*MED23, PTPRB, ZFHX3, TSC1, AXIN2, CDK12, NFE2L2, AHNAK, ACNA1D, MN1, NRG1 BRCA2, IDH2, FGFR2, IRF2, DIS3, TP53, CEP290, RHBDF2*). We identified 19 significant mutant genes for somatic variants in the exon coding region: *MRE11A, ATM, NOTCH2, ATOH8, ASCC1, DOPEY2, HIST2H2AC, APC, ZCWPW1, POU2F3, CTC1, EXOC1, SLC5A12, MEN1, ATP12A, MNX1, SERPINB3, SERPINB4*, and *BCL2*. On this basis, the somatic mutation of the patient was compared with the known driver genes in the database. The possible driver genes in the tumor sample were screened as *ARID1B, MAX, NOTCH2* and *APC* (Fig. 4b), in which a missense mutation of base C instead of base T occurred in the *NOTCH2* gene located at position 120,471,691 on chromosome 1. Finally, 220 differential genes were screened among single nucleotide polymorphisms (SNPs) between tumor tissues and normal control tissues. These 220 genes were classified into 229 functional categories using the Gene Ontology (GO) database, as shown in Fig. 5a, mainly involving biological processes such as biofilm synthesis, cell adhesion, regulation of substance metabolism, regulation of enzyme activity, rRNA processing, and biotransformation. Furthermore, 35 significant pathways related to this tumor were obtained by KEGG pathway enrichment analysis, as shown in Fig. 5b, of which tumor-related routes accounted for 11.4% (4/35), metabolic pathways and other pathways accounted for 25.7% (9/35) and 62.9% (22/35), respectively. Inspired by the patient's lung cancer status during the last follow-up, investigated the microsatellite status. We identified a missense mutation in the exon region of the *PMS2* gene located at 6,026,775 on chromosome 7, in which base C replaced base T.

After exon sequencing, we used IHC technology to verify some genes that showed key changes in the sequencing results. Tumor cell nuclei were strongly positive for TFEB ( +) (Fig. 2a), positive for Melan-A, MSH2 (present +) (Fig. 2k), MLH1 (present +), and PMS2 (present + , focal -), and negative for MSH6 and HMB45.

## Discussion

*TFEB*/6p21/*VEGFA*-amplified RCC defined by the 6p21.1 chromosomal region is a rare and gradually recognized RCC subtype that exists independently of *TFEB*-translocated RCC and has been included in the molecularly defined renal cancer subtypes by the World Health Organization in 2022 [9]. Our knowledge of this tumor is mainly derived from the preliminary studies of Gupta et al. [2–6], and the overall understanding of its biology is minimal. The lack of diagnosis and treatment guidelines makes this tumor challenging to treat, and 40% of cases experience aggressive metastasis or death.

We retrieved 8 papers with complete information about 50 cases of *TFEB*-amplified renal cell carcinoma (Supplementary Table 2) patients whose main characteristics were as follows: (1) Sex: there were 30 cases in males and 20 cases in females, with a male to female ratio of 3:2. (2) Age: the patients’ age ranged from 23 to 80 years, with a mean age of 63.46 and a median age of 65.00. (3) Tumor size: the average tumor size was 8.73 cm. (4) TNM stage: The percentage of TNM stage ≥ pT3 was 30/50. (5) ISUP grading: there was 1 case with a low grade, accounting for 1/40; 3 cases with grade 2, accounting for 3/40; 24 cases with grade 3, accounting for 24/40; 12 cases with grade 4, accounting for 12/40. (6) The presence of distant or regional metastases was confirmed at diagnosis or follow-up: there were 20 cases with complete follow-up information, of which 15 had metastases, representing a metastasis rate of 15/20. (7) Morphological features: microscopically, the tumor cells were morphologically diverse, with cells in nested (12/45), papillary (14/45), pseudopapillary (6/45), tubular papillary (18/45), and clear cell areas (20/45), and such tumors had an overall increase in cytoplasmic eosinophils, accounting for 27/45, some with cell necrosis (7/45). (8) Immunohistochemistry: the analyses revealed positivity for TFEB (+ , 5/7), cathepsin k(+ , 16/27), Melan-A (+ , 28/36) and HMB45 (+ , 6/30) (9) FISH: *TFEB* FISH revealed breaks & GT (10 signals, 32/33); *VEGFA* FISH revealed breaks & GT (10 signals; 14/14). (10) The other molecular genetic features observed were loss of chromosome 3p (6/12), loss of chromosome 7 (2/9), loss of chromosome 17 (4/9), occasional missense mutations in the *SMARCB1* gene, and nonsense mutations in the *FH* gene.

In the 2022 WHO classification of renal cancer, in addition to *TFEB* amplifying renal cancer, *TFEB*-translocated renal cell carcinoma is included, which is a relatively rare subtype of kidney cancer typified by a translocation between the *TFEB* gene on chromosome 6 and the *MALAT1/Alpha* gene on chromosome 11 [10]. In a review of 40 cases of *TFEB* translocated renal cell carcinoma reported in the literature [3, 6, 11, 12] (Supplementary Table 3), combined with the studies of Gupta and Qiuyang Lu et al. [3, 6, 10, 13], we found significant differences between *TFEB* translocation and *TFEB*-amplified tumors in terms of age of disease onset, histological morphology, melanocyte markers, expression of cathepsin k, *VEGFA/CCND3* gene expression, and aggressive behavior. The above differences contribute to the differential diagnosis of the two, as described in detail below (Table 2): 1. Clinicopathological features: there was no noticeable sex difference between the two groups. The former tumor occurred in adults and was small; the latter tumor developed at an older age and occurred in older patients, and the tumor volume was more prominent. 2. Histologic features: both tumors are primarily nonspecific, generally well-defined, and reddish-brown on the cut surface. The typical biphasic histopathological features of "large epithelioid cells and small cells clustered around clear basement membrane-like tissue" are more common in translocated RCC. More extensive morphological features, such as sclerosis and ossification, are occasionally seen in *TFEB*-translocated RCC. Amplified tumors were morphologically diverse, with cytoplasmic eosinophilia (*p* = 0.013) and pseudopapillary, necrotic and true papilla, the characteristics of the amplified tumor. RCC with aberrant *TFEB* expression was a highly graded RCC, and *TFEB*-amplified renal cell carcinoma had a higher proportion of ≥ pT3 in TNM staging (*p* = 0.047). 3. Immunophenotypic features: overexpression of *TFEB* genes frequently drives abnormal expression of melanocyte-associated antigens (HMB45, Melan-A) and osteoblast histone k (cathepsin k); overexpression of cathepsin k (*p* < 0.000), HMB45 (*p* < 0.000), and Melan-A (*p* = 0.028) is more commonly found in *TFEB*-translocated renal cell carcinoma. 4. *TFEB* expression assay: the results of the *TFEB* gene expression assay are correlated with the immunohistochemistry results [2, 3, 6, 14], but at the genetic level, amplified renal tumors have a low tendency to express *TFEB*, which is often accompanied by *VEGFA* gene amplification. Several studies suggest that the low expression of *TFEB* in amplified renal tumors may be attributed to their lack of typical biphasic morphology. 5. Prognosis: translocated RCC had an excellent clinical prognosis with a low recurrence and distant metastasis rate (1/8). Renal tumors with amplifications had a more aggressive clinical course, a higher recurrence and distant metastasis rate (15/20, *p* = 0.004), and a poorer clinical prognosis.

**Table 2:** Summary of the main characteristics of *TFEB* translocation renal cell carcinoma and *TFEB* amplification renal cell carcinoma

Cases		Age (Mean, year)	Sex (Male: female)	Size (Mean, cm)	≥ pT3	Metastasis (follow-up)	Papillary	Tubulopapillary	Pseudopapillary	Morphologic features		Necrosis	Calcification	Pigment	TFEB	IHC(Positivity)			HMB45
										Eosinophilic	Clear Cell					cathepsin k	Melan-A		
*TFEB-*																			
Amplified Tumors TFEB	50	63.46	3:2	8.73	30/50	15/20	14/45	18/45	6/45	27/45	20/45	7/45	/	/	5/7	16/27		28/36	6/30
Translocated Tumors	40	45.6	2:3	7.09	8/24	1/8	5/25	4/25	3/25	7/25	16/25	3/25	3/25	3/25	12/12	36/36		34/35	21/30
*p*		**0.000**	/	0.088	**0.047**	**0.004**	0.406	0.059	1.000	**0.013**	0.140	1.000	/	/	0.123	**0.000**		**0.028**	**0.000**

Statistical analysis was performed by Fisher’s exact test

*Abbreviations*: *IHC* Immunohistochemistry

In sequencing, the CNV mutation in this case was consistent with the already reported by our team [15]. High-frequency CNV analysis yielded diagnostically significant alterations on chromosome 6. The CNV results further suggested that the gain in chromosomes 1q, 2p, 4q, 6p, 16p, 17q, 18q, 19q, 22q and loss in chromosome 18q were consistent with previous findings in *TFEB*-amplified renal cell carcinoma [3, 4, 16]. Nevertheless, the amplification of chromosomes 1p, 4p, 10q, 18q, 19p, and 21p and the loss of chromosome 17q in the present case has not been previously reported.

Subsequently, the germline mutations in this case were analyzed. The susceptibility of *TP53* to mutation in normal tissues adjacent to cancer revealed the instability of the patient's tumor. Single nucleotide polymorphisms (SNPs) between tumor tissues and normal control tissues were analyzed, and the obtained differentially expressed genes were mapped to the KEGG and GO databases. The results are shown in Fig. 5. The results can be interpreted from three levels. First, classical pathways associated with cancer, such as the TGF-β signaling pathway [17, 18] and Hippo signaling pathway [19], were involved. Metabolism-related courses accounted for 25.7%, which was in line with results from previous studies that showed that kidney cancer is a metabolism-driven disease [20]. After enrichment, some pathways were associated with biological dysfunction and abnormal behavior caused by aberrant overexpression of *TFEB* genes, such as E-cadherin, an essential regulator of tumor cell-to-cell interactions, lysosomal biogenesis [21], and autophagy of tumor cells [22, 23]. Given the close correlation between the above partial enrichment pathway and amplified mutations of the *TFEB* gene, which was consistent with our previous CNV results suggesting the presence of *TFEB* amplification, the rationale supporting the diagnosis of *TFEB*-amplified renal cell carcinoma was more robust. Among the genes with somatic missense mutations, *NOTCH2*, *NR3C1*, *NT5E*, *PLAGL1*, and *ACAT2* correlate with the occurrence and development of renal tumors. Among them, the *NOTCH2* gene was related to cell stemness [24], which could induce and regulate the occurrence and apoptosis of tumor cells; *NT5E* could inhibit the growth, EMT process, and AKT/GSK-3β signaling pathway of sunitinib-resistant cells in renal cell carcinoma [25]. It has also been proposed that PLAGL1 protein levels in CCRCC tissues are positively correlated with distant metastasis and worse patient prognosis [26, 27]; the *ACAT2* gene was related to lipid metabolism [28], and its downregulation could lead to a poor tumor-specific survival prognosis. The remaining genes with missense mutations suggest changes associated with cell proliferation and differentiation, amino acid metabolism, nucleotide metabolism, and signal transduction pathways. In this case, a frameshift deletion occurred in the *APC* gene on chromosome 5, which encodes a tumor suppressor protein that acts as an antagonist of the Wnt signaling pathway and is also involved in other processes, including cell migration and adhesion. Transcriptional activation and apoptosis have also been reported in CHRCC metastatic chromophobe renal cell carcinoma with *APC* mutation [26].

During interpreting data, we obtained the diagnosis of *TFEB*-amplified RCC after summarizing the molecular genetic alterations of common and rare subtypes of kidney cancer by the latest guidelines and literature [8, 29]. The development of molecular pathology has constantly advanced our understanding of kidney cancer, and some tumor subtypes based on specific molecular alterations, such as "translocation-associated renal cell carcinoma," were first introduced in the WHO classification in 2004 [7]. However, these molecularly defined tumors have shown a broad morphological spectrum in some recent studies, and whether there is a clear correlation between genotype and phenotype is worth discussing; thus, it is crucial to broaden the idea of differential diagnosis of tumors with the help of molecular tests such as second-generation sequencing [7–9].

During the follow-up, the patient developed poorly differentiated squamous cell carcinoma in the lung one year after kidney cancer surgery; the secondary lung malignancy led us to speculate whether there were some specific alterations at the genetic level in the patient. We first examined tumor mutation burden (TMB) and microsatellite instability (MSI), which are predictors of the efficacy of immune checkpoint inhibitor therapy. The results showed that the TMB was low. Regarding MSI, we first noticed the expression of MMR mismatch repair (MMR) protein and obtained the impact of low expression of MSH6 protein. Meanwhile, seven common loci in MSI were detected by next-generation sequencing technology, and the results suggested that they were microsatellite stable (MMS). However, we found in the exon sequencing results that there was a missense mutation in the exon region of the *PMS2* gene on chromosome [7], in which base C replaced base T. Could the above situation suggest microsatellite instability in this patient? Considering the heterogeneity of the tumor during the assay and the methodology of the assay, the results of this patient's MSI status need to be further discussed and analyzed in the context of the literature.

This patient has multiple tumor characteristics, which was another interesting aspect of this case. Analyzing the expression of genes associated with homologous recombination repair could be beneficial in guiding the patient's clinical treatment. The sequencing results suggested that this patient had a homologous recombination-deficient (HRD) tumor, with the loss of *ATM* and *MRE11A*, which are key genes involved in the process of homologous recombination (HR) repair, suggesting that we could try targeted therapy with poly ADP ribose polymerase (PARP) inhibitors: this patient was relatively sensitive to niraparib (class C), olaparib (class C), rucaparib (class C), and talazoparib (class C). Olaparib, an inhibitor of oral poly ADP-ribose polymerase (PARP), is increasingly being demonstrated in clinical studies to be effective in HRD gene-deficient cell lines, such as those lacking *ATM*, in addition to providing sensitization in combination with chemotherapeutic agents and killing *BRCA1* or *BRCA2* gene-mutated tumor cells. Clinical trials are underway in patients with renal clear cell carcinoma, urothelial carcinoma, and prostate cancer. Talazoparib is a next-generation PARP inhibitor with a dual mechanism of action that stimulates tumor cell death by blocking PARP enzyme activity and binding PARP enzyme to DNA damage sites, and clinical trials of its use in patients with renal clear cell carcinoma are ongoing. The targeting effects of these drugs still need to be explored in depth. Sequencing results can guide targeted dosing, and the loss of these 2 genes may improve the benefit rate of PD-1/PD-L1 inhibitors; thus, this patient may benefit from immunotherapy.

The above findings help explain the complex pathogenesis of lung cancer secondary to kidney cancer two years after the initial patient diagnosis and provide some guidance for the clinical treatment of this disease; unfortunately, the patient developed the disease early and did not have a chance to receive the treatment with relevant drugs. By describing this case, we hope that more patients with a similar disease will have the option to try HRD-related targeted therapy and immunotherapy.

Kidney cancer is a complex disease with unpredictable clinical progression due to typical intertumor and intratumor heterogeneity and high genomic variability [30, 31], which makes it difficult for traditional radiotherapy, chemotherapy, and targeted therapy to overcome the tumor. With the advent of the immune checkpoint inhibitor (ICI) era, a new generation of comprehensive treatment for kidney cancer has emerged [32, 33]. In pre-kidney cancer studies, the mTOR inhibitors everolimus and tesilimus have been approved by the FDA for treating advanced metastatic renal cell carcinoma. These drugs are effective for metastatic TFEB-translocated renal cell carcinoma [34]. Pembrolizumab (Keytruda or pembrolizumab), approved by the US Food and Drug Administration (FDA), is a PD-L1 inhibitor for the treatment of patients with solid tumors, which has brought some clinical benefits to some patients [35–37]. Studies have further shown that *TFEB* affects the biological progression of renal cancer by acting on the mTOR pathway and positively correlates with the expression of PD-L1. In this case, the amplification of *TFEB* and the evaluation of genomic stability provide new opportunities for the combination of targeted therapy and immunotherapy for this type of cancer. Could MSI be a relevant immunotherapeutic marker for kidney cancer treatment? Can mTOR/PARP inhibitors be combined with PD-L1 inhibitors such as pembrolizumab in *TFEB*/6p21/*VEGFA*-amplified RCC? Given the rarity of *TFEB*/6p21/*VEGFA*-amplified renal cell carcinoma, pathologists and clinicians have not reported it domestically or internationally, and the above ideas need to be validated.

Whole-exome molecular genetic analysis of *TFEB*/6p21/*VEGFA*-amplified renal cell carcinoma has enhanced our understanding of this type of tumor. For the first time, we reported possible tumor-related driver genes, alterations in specific chromosomal regions of CNV, and critical genes associated with targeted therapy in *TFEB*/6p21/*VEGFA*-amplified renal cell carcinoma (Table 1), which deepened our understanding of the diagnosis and molecular genetic alterations of *TFEB*/6p21/*VEGFA*-amplified renal cell carcinoma and provided new information for their prognosis and treatment.

### Supplementary Information

**Supplementary Material 1.**

## Data Availability

The datasets used and/or analyzed during the current study are available from the corresponding author on reasonable request.

## Abbreviations

GO: Gene ontology
KEGG: Kyoto Encyclopedia of Genes and Genomes
BP: Biological Process
CC: Cellular Component
MF: Molecular Function
SCNA: Somatic copy number variation
PRCC: Papillary renal cell carcinoma
CCRCC: Clear cell renal cell carcinoma
CHRCC: Chromophobe renal cell carcinoma
ACKD RCC: Acquired cystic kidney disease-associated renal cell carcinoma
TCRCC: Tubulocystic renal cell carcinoma
HLRCC RCC: Hereditary leiomyomatosis and renal cell carcinoma-associated renal cell carcinoma
IHC: Immunohistochemistry
TFEB: Transcription Factor EB
PAX-8: Paired box protein 8
AMACR: A-methylacyl-CoA racemase
CK7: Cytokeratin protein 7
CD10: Cluster of differentiation 10
CD117: Cluster of differentiation 117
CD31: Cluster of differentiation 31
Melan-A: Melanoma antigen recognized by T cell-1
HMB45: Melanoma-related marker
CK20: Cytokeratin 20
CA IX: Carbonic anhydrase IX
SDHB: Succinate dehydrogenase B
MLH1: MutL homolog 1
MSH2: MutS homolog 2
MSH6: MutS homolog 6
PMS2: PMS1 homolog 2
SNP: Single nucleotide polymorphism
InDel: Insertion and deletion
CNV: Copy number variation
SNV: Single nucleotide variant
MSI: Microsatellite instability
MSI-H: Microsatellite instability-high
MMR: Mismatch repair
DMMR: Deficient mismatch repair
HRD: Homologous Recombination Deficiency
PARP: Poly ADP ribose polymerase
ICI: Immune checkpoint inhibitor
TMB: Tumor mutation burden
HLA-1: Human leukocyte antigen class 1
FISH: Fluorescence in situ hybridization
