# Supplementary material for: A rare case of TFEB/6p21/VEGFA-amplified renal cell carcinoma diagnosed by whole-exome sequencing: clinicopathological and genetic feature report and literature review
Source: Diagn Pathol. 2024 May 10;19:66. doi: 10.1186/s13000-024-01476-3 (PMC11084048; doi:10.1186/s13000-024-01476-3)
Supplement: Supplementary file 1 — Supplementary Material 1. [file 13000_2024_1476_MOESM1_ESM.docx]

Supplementary Table 1 Summary of global mutations in the molecular genetics of this patient.

|  | Sample | | CDS | | | Synonymous_SNP | | | Missense  _SNP | | | | stopgain | | | stoploss | | | unknown | | intronic | | | | UTR3 | | | UTR5 | | | | splicing | | | ncRNA_  exonic | | | | ncRN_  intronic | | | ncRNA_  splicing | | | upstream | | | | downstream | | | | intergenic | | | Others | | Total | |
| --- | --- | --- | --- | --- | --- | --- | --- | --- | --- | --- | --- | --- | --- | --- | --- | --- | --- | --- | --- | --- | --- | --- | --- | --- | --- | --- | --- | --- | --- | --- | --- | --- | --- | --- | --- | --- | --- | --- | --- | --- | --- | --- | --- | --- | --- | --- | --- | --- | --- | --- | --- | --- | --- | --- | --- | --- | --- | --- | --- |
| SNP | N | | 20911 | | | 10845 | | | 9585 | | | | 70 | | | 11 | | | 400 | | 61624 | | | | 3452 | | | 1738 | | | | 492 | | | 1868 | | | 3243 | | | | 17 | | | 1264 | | | | 565 | | | | 7964 | | | 99 | | 103237 | |
|  | T | | 19981 | | | 10365 | | | 9163 | | | | 69 | | | 10 | | | 374 | | 51676 | | | | 2912 | | | 1454 | | | | 480 | | | 1672 | | | 2697 | | | | 18 | | | 991 | | | | 456 | | | | 6943 | | | 68 | | 89348 | |
|  | | Sample | | CDS | Frameshift  deletion | | | Frameshift  insertion | | | | Nonframeshitdeletion | | | Nonframeshift  insertion | | | stopgain | | stoploss | | | unknown | | | | intronic | | | | UTR3 | | UTR5 | | | splicing | | | | ncRNA  exonic | | | ncRNA  intronic | ncRNA  splicing | | | | upstream | | | downstream | | | intergenic | | | Others | | Total |
| INDEL | | N | | 499 | 71 | | | 49 | | | | 168 | | | 134 | | | 3 | | 0 | | | 74 | | | | 8753 | | | | 504 | | 194 | | | 140 | | | | 164 | | | 397 | 6 | | | | 166 | | | 82 | | | 941 | | | 12 | | 11858 |
|  |  | T | | 469 | 78 | | | 45 | | | | 163 | | | 107 | | | 3 | | 0 | | | 73 | | | | 6842 | | | | 394 | | 150 | | | 119 | | | | 139 | | | 306 | 5 | | | | 116 | | | 57 | | | 769 | | | 9 | | 9375 |
|  | | Sample | | CDS | Synonymous_SNP | | | | | | Missense_SNP | | | | stopgain | | stoploss | | | unknown | | | intronic | | | UTR3 | | | | UTR5 | | | splicing | | | | ncRNA_exonic | | | | | | ncRNA_intronic | | | | upstream | | | downstream | | | | intergenic | | | Others | | Total |
| SNP | | T | | 205 | 77 | | | | | | 114 | | | | 10 | | 1 | | | 3 | | | 517 | | | 41 | | | | 7 | | | 10 | | | | 12 | | | | | | 32 | | | | 6 | | | 5 | | | | 72 | | | 2 | | 909 |
|  | | Sample | | CDS | | | Frameshift _deletion | | | | | frameshift _insertion | | | | | Nonframeshift _deletion | | | | | | | intronic | | | | | UTR3 | | | | | ncRNA _intronic | | | | | | | upstream | | | | | downstream | | | | | | intergenic | | | Total | | | | |
| INDEL | | T | | 10 | | | 5 | | | | | 2 | | | | | 3 | | | | | | | 22 | | | | | 1 | | | | | 3 | | | | | | | 1 | | | | | 1 | | | | | | 1 | | | 39 | | | | |
|  | | Sample | | Gain_count | | | | | | Gain_size | | | | Loss_count | | | | Loss_size | | | | Total_count | | | | | | | | | Total_size | | | | | | | | | | | | | | | | | | | | | | | | | | | | |
| CNV | | T | | 76 | | | | | | 262470597 | | | | 14 | | | | 141673530 | | | | 90 | | | | | | | | | 404144127 | | | | | | | | | | | | | | | | | | | | | | | | | | | | |

Abbreviations: CDS, coding sequence region; SNP, single nucleotide polymorphism; INDEL, Insertion and deletion; CNV, Copy number variation.

Supplementary Table 2 Clinical and pathological characteristics of 50 patients with *TFEB* amplification renal cell carcinoma.

| Case | Case References | Age | Gender | Size | Stage TNM | ISUP  grade | Follow-up  （month） | Metastasis | Morphologic features |
| --- | --- | --- | --- | --- | --- | --- | --- | --- | --- |
| 1 | Peck ova et al | 77 | F | 12 | pT3NxM1 | 4 | 2.5 | Adrenal gland and lung metastasis | Nested/Pseudopapillary and papillary eosinophilic cells; with necrosis |
| 2 | Durinck et al | 56 | M | NA | NA | Low | NA | NA | Papillary architecture and oncocytic features |
| 3 | Argani et al | 64 | F | 10.9 | pT3bN2Mx | 3 | 28 | NA | Nested/Pseudopapillary architecture with eosinophilic cells ;with necrosis |
| 4 | Argani et al | 71 | M | 3 | pT3aNxMx | 3 | NA | Periaortic lymph node metastasis | Papillary architecture with clear and eosinophilic cells; psammoma; focal necrosis bodies |
| 5 | Argani et al | 65 | M | 1.9 | pT1NxMx | 3 | NA | NA | Nested/Pseudopapillary architecture with eosinophilic cells; focal necrosis |
| 6 | Argani et al | 23 | F | 7 | pT2xMx | 4 | NA | Vaginal metastasis | Nested/Pseudopapillary with eosinophilic cells; papillary with psammoma bodies; focal biphasic pattern; with necrosis |
| 7 | Argani et al | 77 | F | 4 | pT1NxMx | 3 | NA | NA | Nested/Pseudopapillary with eosinophilic cells; with necrosis |
| 8 | Argani et al | 78 | F | 12 | pT3bNxMx | 3 | NA | NA | Nested/Pseudopapillary with eosinophilic cells; with necrosis |
| 9 | Williamson et al | 57 | M | 19.5 | pT3aN0Mx | NA | NA | NA | Clear cell and eosinophilic tubulopapillary, prominent nucleoli |
| 10 | Williamson et al | 62 | F | 12.5 | pT3aNx Mx | NA | NA | NA | Clear cell and eosinophilic tubulopapillary, chromophobe like areas |
| 11 | Williamson et al | 78 | F | 4.3 | pT3aNx Mx | NA | NA | NA | Eosinophilic papillary and chromophobe like with calcifications |
| 12 | Gupta et al | 34 | M | 9 | pT3cNxM1 | 3 | 21 | Soft tissue metastasis | High-grade papillary, oncocytic, focal areas with clear cell morphology |
| 13 | Gupta et al | 80 | M | 1.8 | pT1aNxM0 | 3 | 47 | - | High-grade tubulopapillary, oncocytic |
| 14 | Gupta et al | 65 | F | 9.5 | pT3aNxM0 | 3 | 73 | Soft tissue metastasis | High-grade tubulopapillary, oncocytic |
| 15 | Gupta et al | 69 | F | 2.5 | pT1aNxM0 | 2 | 265 | - | Intermediate-grade, clear cell, oncocytic and tubulopapillary, with biphasic features |
| 16 | Gupta et al | 78 | M | 5.5 | pT3cNxM0 | 3 | 254 | - | High-grade tubulopapillary, oncocytic, with biphasic features |
| 17 | Gupta et al | 62 | M | 13 | pT2bNxM0 | 2 | 194 | - | Intermediate grade, clear cell and oncocytic |
| 18 | Gupta et al | 70 | M | 10 | pT2aNxM0 | 4 | 18 | Bone and lung metastasis | High-grade、solid、cystic and tubulopapillary; oncocytic with voluminous cytoplasm; Tubulopapillary: clear cell and oncocytic areas. Papillary areas with luminal clear cell features |
| 19 | Gupta et al | 83 | M | 6.5 | pT3cNxM1 | 4 | 40 | Brain, lung, adrenal gland and lymph-nodes | High-grade tubulopapillary, oncocytic |
| 20 | Gupta et al | 56 | M | 13 | pT3aNxM0 | 4 | 242 | NA | High-grade tubulopapillary, oncocytic focally biphasic; areas with clear cell change |
| 21 | Gupta et al | 73 | M | 3.7 | pT3aNxMx | 3 | NA | NA | High-grade tubulopapillary, oncocytic, focal nested growth pattern |
| 22 | Gupta et al | 68 | F | 18.5 | pT3cN0Mx | 3 | 18 | Bone and soft tissue metastasis | NA |
| 23 | Skala et al | 68 | F | 6.5 | pT3aNxMx | 3 | NA | NA | Nested/papillary; Dual (eosinophilic and clear) cytoplasmic tones |
| 24 | Skala et al | 65 | M | 5.5 | pT3aNxMx | 3 | NA | NA | Dual (eosinophilic and clear) cytoplasmic tones、clear cell RCC |
| 25 | Skala et al | 48 | F | 10.1 | pT2bNxMx | 4 | NA | NA | Papillary; dual (eosinophilic and clear) cytoplasmic tones |
| 26 | Skala et al | 68 | M | 12.2 | pT4N1M1 | 4 | NA | NA | Nested; dual (eosinophilic and clear) cytoplasmic tones、clear cell RCC、 |
| 27 | Skala et al | 72 | M | 7 | pT3aNxMx | 3 | NA | NA | Papillary、eosinophilic |
| 28 | Skala et al | 69 | M | 5.9 | pT3aN1Mx | 3 | NA | NA | Papillary; dual (eosinophilic and clear) cytoplasmic tones |
| 29 | Mendel et al | 55 | F | 8 | pT2aN0M0 | 4 | 161 | Lung metastasis | Clear and eosinophilic cells, with large and irregular nuclei; Clear or granular eosinophilic  cytoplasm was abundant. |
| 30 | Mendel et al | 55 | F | 17 | pT4NxM1 | 4 | 1 | Renal vein thrombus brain metastasis | Sarcomatoid RCC |
| 31 | Mendel et al | 60 | M | 14 | pT3cN0M1 | 4 | 14 | Renal vein thrombus, liver metastasis | Unclassified RCC |
| 32 | Caliò et al | 69 | M | 7 | pT2aNxMx | 3 | 14 | NA | NA |
| 33 | Caliò et al | 41 | F | 3 | pT1aNxMx | 2 | NA | NA | NA |
| 34 | Caliò et al | 79 | M | 10 | pT2aNxMx | 3 | NA | NA | NA |
| 35 | Gupta et al | 64 | M | 2 | pT1aNxMx | 3 | NA | NA | Tubulopapillary、Eosinophilic |
| 36 | Gupta et al | 61 | F | NA | pT3aNx Mx | 4 | NA | NA | Tubulopapillary、Eosinophilic |
| 37 | Gupta et al | 53 | M | 8.5 | pT3aNx M0 | 4 | NA | + | Nested、Clear |
| 38 | Gupta et al | 68 | M | 13 | pTxNxMx | 3 | NA | NA | Tubulopapillary、Eosinophilic |
| 39 | Gupta et al | 66 | F | 14 | pT3aNx Mx | 3 | NA | NA | Nested、Clear |
| 40 | Gupta et al | 73 | F | 14.4 | pTxNxMx | 3 | NA | NA | Nested、Eosinophilic、Solid |
| 41 | Gupta et al | 73 | M | 5 | pT1bNxM0 | 3 | NA | - | Tubulopapillary、Eosinophilic |
| 42 | Gupta et al | 61 | M | 13 | pT2bNxM1 | 3 | NA | + | papillary、Eosinophilic |
| 43 | Gupta et al | 58 | M | 9.1 | pT3bN1M1 | NA | NA | + | NA |
| 44 | Gupta et al | 59 | M | 9.2 | pT2aN1Mx | NA | NA | + | Eosinophilic papillary, broad papillae |
| 45 | TCGA-BQ-7048-01A | 64 | M | 11 | pT3aN0Mx | NA | NA | NA | Clear cell and tubulopapillary |
| 46 | TCGA-UZ-A9PQ-01A | 59 | M | 9.2 | Pt3aN1Mx | 3 | NA | NA | Eosinophilic |
| 47 | TCGA-B9-A69E-01A | 71 | M | 8 | pT3aNxMx | NA | NA | NA | Oncocytic and basophilic papillary, focal clearing |
| 48 | TCGA-GL-7966-01A | 28 | F | 6.5 | pT3aN1Mx | NA | NA | NA | Eosinophilic tubulopapillary, prominent nucleoli, desmoplastic |
| 49 | TCGA-Q2-A5QZ-01A | 61 | F | 5.3 | pT3aNxMx | NA | NA | NA | Eosinophilic tubulopapillary, prominent nucleoli, focal clearing |
| 50 | TCGA-A3-3313-01 | 59 | M | 4.5 | pT1bNxMx | NA | NA | NA | Clear cell and tubulopapillary |

Supplementary Table 2 Immunohistochemical and molecular characteristics of 50 patients with *TFEB* amplification renal cell carcinoma.

| Case | Case References | IHC | | | | TFEB FISH | VEGFA FISH | loss3p | gain 7 | gain 17 | Other  Quantitative  anomalies | Other related  Genes |
| --- | --- | --- | --- | --- | --- | --- | --- | --- | --- | --- | --- | --- |
|  |  | TFEB | Cathepsin K | Melan-A | HMB45 |  |  |  |  |  |  |  |
| 1 | Peckova et al | NA | + | + | + | Amplification | NA | NA | NA | NA | NA | NA |
| 2 | Durinck et al | NA | NA | NA | NA | NA | NA | NA | NA | NA | NA | NA |
| 3 | Argani et al | +++ | - | + | - | >10 signals | NA | NA | NA | NA | NA | NA |
| 4 | Argani et al | - | - | + | - | >10 signals | NA | NA | NA | NA | NA | NA |
| 5 | Argani et al | +++ | + | + | - | >10 signals | NA | NA | NA | NA | NA | NA |
| 6 | Argani et al | + | + | + | + | >10 signals | NA | NA | NA | NA | NA | NA |
| 7 | Argani et al | +++ | + | + | + | >10 signals | NA | NA | NA | NA | NA | NA |
| 8 | Argani et al | + | + | + | - | >10 signals | NA | NA | NA | NA | NA | NA |
| 9 | Williamson et al | NA | + | + | NA | >10 signals | NA | No | NA | NA | NA | NA |
| 10 | Williamson et al | NA | - | - | - | >10 signals | NA | Yes (VHL induced) | NA | NA | NA | NA |
| 11 | Williamson et al | NA | + | + | NA | >10 signals | NA | No | NA | NA | NA | NA |
| 12 | Gupta et al | NA | NA | + | - | >10 signals | >10 signals | NA | NA | NA | NA | NA |
| 13 | Gupta et al | NA | NA | - | - | >10 signals | >10 signals | NA | NA | NA | NA | NA |
| 14 | Gupta et al | NA | NA | - | - | >10 signals | >10 signals | NA | NA | NA | NA | NA |
| 15 | Gupta et al | NA | NA | + | - | >10 signals | >10 signals | NA | NA | NA | NA | NA |
| 16 | Gupta et al | NA | NA | + | - | >10 signals | >10 signals | NA | NA | NA | NA | NA |
| 17 | Gupta et al | NA | NA | + | + | >10 signals | >10 signals | NA | NA | NA | NA | NA |
| 18 | Gupta et al | NA | NA | + | - | >10 signals | >10 signals | NA | NA | NA | NA | NA |
| 19 | Gupta et al | NA | NA | + | - | >10 signals | >10 signals | NA | NA | NA | NA | NA |
| 20 | Gupta et al | NA | NA | - | - | >10 signals | >10 signals | NA | NA | NA | NA | NA |
| 21 | Gupta et al | NA | + | NA | NA | >10 signals | >10 signals | NA | NA | NA | NA | NA |
| 22 | Gupta et al | NA | NA | + | - | >10 signals | >10 signals | NA | NA | NA | NA | NA |
| 23 | Skala et al | NA | + | + | NA | NA | NA | NA | NA | NA | NA | NA |
| 24 | Skala et al | NA | + | NA | NA | NA | NA | NA | NA | NA | NA | NA |
| 25 | Skala et al | NA | - | + | NA | NA | NA | NA | NA | NA | NA | NA |
| 26 | Skala et al | NA | - | + | - | NA | NA | NA | NA | NA | NA | NA |
| 27 | Skala et al | NA | - | + | NA | NA | NA | NA | NA | NA | NA | NA |
| 28 | Skala et al | NA | - | + | NA | NA | NA | NA | NA | NA | NA | NA |
| 29 | Mendel et al | NA | - | - | - | >20 signals | NA | No | No | No | +2q, +5p, +6p / -1p, -2q, -5p, -6p, -8q, -13q | NA |
| 30 | Mendel et al | NA | - | - | - | 4-10 signals | NA | No | Partial | Partial | +1p, +4p, +5p, +6p, +7p, +8q, +10p, +10q, +11p, +11q, +12p, +14q, +17p, +20,+22q / -1q, -2q, -3p, -4p, -7q, -8p, -9p, -10p, -10q, -13q,-14q, -16p, -18p, -X | NA |
| 31 | Mendel et al | NA | - | + | - | 10-20 signals | NA | Yes (VHL induced) | No | No | +4p, +6q, +8 / -1p, -3, -4p, -6p, -p9, -10, -11p, -13, -15, -17q, -Y | NA |
| 32 | Caliò et al | NA | + | + | - | Break+>10 signals | >10 signals | NA | NA | NA | NA | NA |
| 33 | Caliò et al | NA | + | + | + | >10 signals | >10 signals | NA | NA | NA | NA | NA |
| 34 | Caliò et al | NA | + | - | - | >10 signals | >10 signals | NA | NA | NA | NA | NA |
| 35 | Gupta et al | NA | + | + | - | NA | NA | NA | NA | NA | NA | NA |
| 36 | Gupta et al | NA | + | + | - | NA | NA | NA | NA | NA | NA | NA |
| 37 | Gupta et al | - | NA | NA | NA | NA | NA | NA | NA | NA | NA | NA |
| 38 | Gupta et al | NA | + | + | + | NA | NA | NA | NA | NA | NA | NA |
| 39 | Gupta et al | NA | - | - | - | NA | NA | NA | NA | NA | NA | NA |
| 40 | Gupta et al | NA | NA | + | - | NA | NA | NA | NA | NA | NA | NA |
| 41 | Gupta et al | NA | NA | NA | NA | NA | NA | NA | NA | NA | NA | NA |
| 42 | Gupta et al | NA | NA | NA | NA | NA | NA | NA | NA | NA | NA | NA |
| 43 | Gupta et al | NA | NA | NA | NA | NA | NA | NA | NA | NA | NA | NA |
| 44 | Gupta et al TCGA | NA | NA | NA | NA | NA | NA | NA | NA | NA | NA | NA |
| 45 | TCGA-BQ-7048-01A | NA | NA | NA | NA | >10 signals | NA | No | No | No | -12, -19^ | SMARCB1 missense |
| 46 | TCGA-UZ-A9PQ-01A | NA | NA | NA | NA | >10 signals | NA | Yes (VHL induced) | No | No | +2q, +6p, +10p / -1p, -2p, -3p, -4p, -6p, -9p, -10p, -11q, -18, -20, -22^ | NA |
| 47 | TCGA-B9-A69E-01A | NA | NA | NA | NA | >10 signals | NA | Yes (VHL induced) | No | No | +2q, +6p / -1p, -2p, -3p, -6p, -9^ | NA |
| 48 | TCGA-GL-7966-01A | NA | NA | NA | NA | >10 signals | NA | No | No | Yes | +1q, +2, +6q, +8, +11, +12, +14, +16, +17, +20 / -1p, -4, -5p, -5q, -9p, -10, -13, -15, -18, -21q, -22q | FH nonsense(somatic) |
| 49 | TCGA-Q2-A5QZ-01A | NA | NA | NA | NA | >10 signals | NA | Yes (VHL induced) | Partial | Yes | +2q, +6p, +7p, +8, +17 / -1p, -2q, -3p, -4, -5p, -6p, -13p, -14, -15p, -17p, -18, -19,-22,-X | NA |
| 50 | TCGA-A3-3313-01 | NA | NA | NA | NA | >10 signals | NA | Yes (VHL induced) | No | Yes | +6p, +17, +20 / -1p, -3p, -6p, -18^ | NA |

Abbreviations: IHC, Immunohistochemistry; TCGA, The Cancer Genome Atlas; NA, not applicable.

Supplementary Table 3 Clinicopathological and molecular characteristics of 40 patients with *TFEB* translocation renal cell carcinoma.

| **Case** | **Case References** | **Age** | **Gender** | **Size** | **Stage**  **TNM** | **ISUP grade** | **Follow-up（month）** | **metastasis** | **Morphologic**  **features** | **IHC** | | | | **TFEB FISH** | **Genetic background** |
| --- | --- | --- | --- | --- | --- | --- | --- | --- | --- | --- | --- | --- | --- | --- | --- |
|  |  |  |  |  |  |  |  |  |  | TFEB CathepsinK Melan-A HMB45 | | | |  |  |
| 1 | Gupta et al | 51 | F | 14.2 | pT3NxMx | 3 | NA |  | Tubulopapillary、Eosinophilic、Necrosis | NA | **+** | **+** | **+** | NA | NA |
| 2 | Gupta et al | 12 | M | 5.4 | pT1bN0M0 | 3 | + | - | Acinar、Clear | **+** | **+** | NA | NA | NA | NA |
| 3 | Gupta et al | 78 | F | 3.5 | pT1aN0M0 | 3 | + | - | Solid、Clear | **+** | **+** | NA | NA | NA | NA |
| 4 | Gupta et al | 49 | F | 2 | pTxNxMx | 2 | NA |  | Nested、Clear | NA | **+** | **+** | **-** | NA | NA |
| 5 | Gupta et al | 38 | F | 5.3 | pTxNxMx | 2 | NA |  | Acinar、Clear | NA | **+** | **+** | **+** | NA | NA |
| 6 | Gupta et al | 59 | F | 1.9 | pTxNxMx | 2 | NA |  | Nested、Clear、Calcification | NA | **+** | **+** | **-** | NA | NA |
| 7 | Gupta et al | 72 | F | 4 | pT3aNxMx | 4 | NA |  | Tubulopapillary、Eosinophilic、Sarcomatoid、Pigment | **+** | **+** | **+** | **+** | NA | NA |
| 8 | Gupta et al | 60 | F | 4.5 | pT1bNxMx | 2 | NA |  | Tubulopapillary、Eosinophilic | **+** | **+** | NA | NA | NA | NA |
| 9 | Gupta et al | 68 | M | 2.8 | pT1aNxMx | 2 | NA |  | Solid、Clear | **+** | **+** | + | NA | NA | NA |
| 10 | Gupta et al | 55 | M | 9.5 | pT3bNxM1 | 3 | **+** | **+** | Nested、Clear、Necrosis | **+** | **+** | NA | NA | NA | NA |
| 11 | Gupta et al | 23 | F | 3.5 | pT1NxMx | 4 | NA |  | Nested、Clear、Rhabdoid | NA | **+** | **+** | **+** | NA | NA |
| 12 | Gupta et al | 35 | F | NA | pTxNxMx | 1 | NA |  | Other、Clear | NA | **+** | **+** | NA | NA | NA |
| 13 | Gupta et al | 61 | F | 5 | pTxNxMx | 2 | NA |  | Tubulopapillary、Clear、Necrosis | NA | **+** | NA | NA | NA | NA |
| 14 | Gupta et al | 23 | F | NA | pTxNxMx | 2 | NA |  | Acinar、Clear | NA | **+** | **+** | NA | NA | NA |
| 15 | Gupta et al | 51 | M | 14.6 | pTxNxMx | 2 | NA |  | Solid、Clear、Calcification | NA | **+** | **+** | **-** | NA | NA |
| 16 | Gupta et al | 24 | M | 6.4 | pT1bNxMx | 1 | NA |  | Solid、Clear | NA | **+** | **+** | NA | NA | NA |
| 17 | Gupta et al | 43 | F | 10.3 | pTxNxMx | 1 | NA |  | Nested、Clear、Pigment、Calcification | NA | **+** | **+** | + | NA | NA |
| 18 | Gupta et al | 41 | F | 5.8 | pT1bNxMx | 2 | **+** | **-** | Solid、Clear | **+** | **+** | **+** | NA | NA | NA |
| 19 | Caliò et al | 55 | M | 3 | NA | NA | NED 78 months | NA | NA | NA | **+** | **+** | **+** | break 65% | NEAT1-TFEB |
| 20 | Caliò et al | 81 | F | 6.5 | NA | NA | NED 12 months | NA | NA | NA | **+** | **+** | **+** | break 91% | ACTB-TFEB |
| 21 | Caliò et al | 54 | F | 7 | NA | NA | NED 36 months | NA | NA | NA | **+** | **+** | **+** | break 80% | MALAT1-TFEB |
| 22 | Caliò et al | 20 | F | 9.5 | NA | NA | NED 36 months | NA | NA | NA | **+** | **+** | **+** | break 75% | MALAT1-TFEB |
| 23 | Caliò et al | 40 | F | 14 | NA | NA | AWD 24 months | NA | NA | NA | **+** | + | **+** | break 85% | MALAT1-TFEB |
| 24 | Caliò et al | 69 | M | 10 | NA | NA | NED 6 months | NA | NA | NA | **+** | **+** | **+** | break 70% | MALAT1-TFEB |
| 25 | Caliò et al | 64 | F | 11 | NA | NA | NED 13 months | NA | NA | NA | **+** | **+** | **+** | break 58% | MALAT1-TFEB |
| 26 | Caliò et al | 19 | F | 5.5 | NA | NA | NED 2 months | NA | NA | NA | **+** | **+** | **+** | break 74% | MALAT1-TFEB |
| 27 | Caliò et al | 34 | M | 7 | NA | NA | NED 30 months | NA | NA | NA | **+** | **+** | **+** | break 78% | MALAT1-TFEB |
| 28 | Caliò et al | 42 | F | 10 | NA | NA | DOD 46 months | NA | NA | NA | **+** | **+** | **+** | break 94% | MALAT1-TFEB |
| 29 | Caliò et al | 33 | M | 8 | NA | NA | AWD 48 months | NA | NA | NA | **+** | **+** | **-** | break 61% | MALAT1-TFEB |
| 30 | Caliò et al | 73 | M | 16 | NA | NA | NED 1 months | NA | NA | NA | **+** | **+** | **+** | break 80% | MALAT1-TFEB |
| 31 | Caliò et al | 69 | M | 7 | NA | NA | AWD 14 months | NA | NA | NA | **+** | **+** | **-** | break 80% | NOt Found |
| 32 | Ge et al | 32 | M | 12 | NA | NA | 7 months | **-** | Eosinophilic、Clear、Pigment | NA | NA | **+** | **+** | break 64% | NA |
| 33 | Ge et al | 21 | F | 3 | NA | NA | 2 months | **-** | NA | NA | NA | **+** | **+** | break 66% | NA |
| 34 | Ge et al | 66 | F | 2.5 | NA | NA | 9 months | **-** | NA | NA | NA | **-** | **-** | break 16% | NA |
| 35 | Wyvekens  et al | 16 | F | 6.4 | NA | 2 | 15 months | **-** | Solid、biphasic "rosette-like" pattern | NA | NA | **+** | **+** | break 51% | MALAT1-TFEB |
| 36 | Kuroda et al | 17 | F | 11 | pT3aN0M0 | 3 | NA | NA | Pseudorosette、Psammoma bodies、Papillary、 "rosette-like" pattern、Eosinophilic | **+** | **+** | **+** | **+** | NA | NA |
| 37 | Kuroda et al | 45 | M | 6 | pT3aN1M1 | 4 | DOD 28 months | NA | Pseudorosette、Psammoma bodies、Papillary、 "rosette-like" pattern | **+** | **+** | **+** | **+** | NA | NA |
| 38 | Kuroda et al | 40 | M | 9 | pT2N0M0 | 2 | AWOD 84 months | NA | Pseudorosette、Psammoma bodies、Papillary、 "rosette-like" pattern | **+** | **+** | **+** | **-** | NA | NA |
| 39 | Kuroda et al | 57 | M | 2.8 | pT1aN0M0 | 1 | DOAD 19 months | NA | Eosinophilic、Papillary | **+** | **+** | **+** | **-** | break 42% | NA |
| 40 | Kuroda et al | 34 | M | 3.5 | pT1aN0M0 | 1 | NA | NA | Pseudorosette、Papillary、 "rosette-like" pattern、Eosinophilic | **+** | **+** | **+** | **-** | break 62.5% | NA |

Abbreviations: F, female; M, male; NA, data not available; TNM, Tumor Node Metastasis; ISUP, International Society of Urological Pathology.
